# Cardiac Morphology and Function, and Blood Gas Transport in Aquaporin-1 Knockout Mice

**DOI:** 10.3389/fphys.2016.00181

**Published:** 2016-05-24

**Authors:** Samer Al-Samir, Yong Wang, Joachim D. Meissner, Gerolf Gros, Volker Endeward

**Affiliations:** ^1^Abteilung Molekular- und Zellphysiologie, AG Vegetative Physiologie 4220, Medizinische Hochschule HannoverHannover, Germany; ^2^Division Molecular and Translational Cardiology, Department Cardiology and Angiology, Medizinische Hochschule HannoverHannover, Germany

**Keywords:** aquaporin-1, blood gases, heart morphology, knockout mice, Pressure-Volume-loop technique

## Abstract

We have studied cardiac and respiratory functions of aquaporin-1-deficient mice by the Pressure-Volume-loop technique and by blood gas analysis. In addition, the morphological properties of the animals' hearts were analyzed. In anesthesia under maximal dobutamine stimulation, the mice exhibit a moderately elevated heart rate of < 600 min^−1^ and an O_2_ consumption of ~0.6 ml/min/g, which is about twice the basal rate. In this state, which is similar to the resting state of the conscious animal, all cardiac functions including stroke volume and cardiac output exhibited resting values and were identical between deficient and wildtype animals. Likewise, pulmonary and peripheral exchange of O_2_ and CO_2_ were normal. In contrast, several morphological parameters of the heart tissue of deficient mice were altered: (1) left ventricular wall thickness was reduced by 12%, (2) left ventricular mass, normalized to tibia length, was reduced by 10–20%, (3) cardiac muscle fiber cross sectional area was decreased by 17%, and (4) capillary density was diminished by 10%. As the P-V-loop technique yielded normal end-diastolic and end-systolic left ventricular volumes, the deficient hearts are characterized by thin ventricular walls in combination with normal intraventricular volumes. The aquaporin-1-deficient heart thus seems to be at a disadvantage compared to the wild-type heart by a reduced left-ventricular wall thickness and an increased diffusion distance between blood capillaries and muscle mitochondria. While under the present quasi-resting conditions these morphological alterations have no consequences for cardiac function, we expect that the deficient hearts will show a reduced maximal cardiac output.

## Introduction

AQP1 has first been reported to act as a membrane water channel by Preston et al. (1992), replacing the long-standing idea that water passes biological membranes through their lipid phases. Later on, several additional functions of AQP1 have been established, among them its function as a channel for CO_2_ (Nakhoul et al., 1998; Endeward et al., 2006) and for NH_3_ (Nakhoul et al., 2001; Musa-Aziz et al., 2009). Moreover, permeabilities of the AQP1 channel have been postulated for hydrogen peroxide (Almasalmeh et al., 2014), for nitric oxide (Herrera et al., 2006), and for oxygen (Wang et al., 2007). During the last 10 years, evidence has been accumulated that AQP1 plays a crucial role in angiogenesis and in general in cell migration (Saadoun et al., 2005).

The present study was prompted by three recent observations: (a) Montiel et al. (2014) reported a reduced weight of the hearts of AQP1-deficient mice compared to normal mice; (b) it has been reported that the spontaneous physical activity of AQP1-deficient mice on running wheels is 40% lower than that of WT mice (Boron, 2010); (c) we have observed that the maximal metabolic rate of AQP1-knockout mice is significantly lower in AQP1-knockout than in WT mice (Al-Samir et al., submitted for publication). We have asked therefore, whether a reduced cardiac output could be the consequence of observation a and cause observations b and c. We have therefore undertaken the following measurements on the hearts of AQP1-deficient mice:

1. We studied for the first time cardiac function of AQP1-KO and WT mice by the P-V loop technique. This had to be done in anesthesia implying that hearts were not maximally activated and results were largely representative of animals' resting conditions.
2. Furthermore, we performed a detailed study of the morphology of the hearts of these mice, with the intention to see whether their morphology suggests restraints imposed on maximal cardiac performance.
3. In addition we present for the first time comprehensive blood gas data characterizing pulmonary and peripheral gas exchange in these mice under anesthesia, i.e., under conditions close to resting conditions. This was expected to indicate whether lack of AQP1 leads to a functionally significant impairment of pulmonary or peripheral gas exchange under resting conditions.

The major findings of this paper are, firstly, that AQP1-deficient mice have thinner ventricle walls and poorer capillarisation of cardiac muscle than normal mice. Secondly, the AQP1-deficient mice exhibit normal cardiac functions, e.g., normal stroke volume and cardiac output. Thirdly, these animals show, under the conditions just mentioned, normal pulmonary, and peripheral exchange of O_2_ and CO_2_. The second and third groups of findings explain why the AQP1-KO animals under laboratory conditions show no pathological cardiac or respiratory symptoms whatsoever.

However, the thin ventricle walls that go along with a reduced cardiac muscle mass suggest that under conditions of maximal workload these hearts will generate a reduced cardiac output compared to wildtype hearts. Thus, maximal exercise capacity of the KO animals is expected to be reduced. Regarding the cause of the reduced cardiac muscle mass, we speculate that lack of AQP1 leads to a reduced capillarisation and that this causes an inhibition of the growth of cardiac muscle fibers.

## Methods

### Animals and pressure-volume loop measurements

Breeding pairs of heterozygous aquaporin-1 ko mice were kindly provided by Dr. Alan S. Verkman (San Francisco, USA; Ma et al., 1998; mice bred on a BL6 background). They were intercrossed to obtain homozygous AQP1-KO mice and WT littermate controls, as ascertained by PCR genotyping using the DNA from tail snippets and specific primers (AAG TCA ACC TCT GCT CAG CTG GG for AQP1-WT, CTC TAT GGC TTC TGA GGC GGA AAG for AQP1 Neo, and ACT CAG TGG CTA ACA ACA AAC AGG for AQP1-KO, in one single PCR reaction). The mice used in the study had an average age of 18 weeks (SD ± 4.7, *n* = 14). Male and female groups were age-matched.

*In vivo* pressure-volume (P-V) loop measurements were conducted under 2% isoflurane anesthesia. A 1.4 F micromanometer conductance catheter (SPR-839, Millar Instruments, Houston, TX) was inserted via the right carotid artery and steady-state P-V loops were sampled at a rate of 1 kHz. In each animal, P-V loops were measured sequentially under infusion via a second catheter inserted into the jugular vein of 0, 2.5, 5, 10, 20, and 40 ng dobutamine/g body weight/min (every dose for 3 min). P-V loops were analyzed by a sample-blinded investigator (Y.W). At the end of the P-V loop recordings, and immediately after a final infusion pulse of 40 ng dobutamine/g/min, left and right ventricle were punctured to obtain arterial and mixed-venous blood gas samples. Animals were immediately killed thereafter and organs were harvested for further analysis. All animal procedures were approved by the local state authorities.

### Blood gas analyses

0.3 ml blood samples were taken by puncture from each LV and RV of all mice used for P-V loop measurements. This was done immediately before termination of the animal experiment and under final stimulation by a repeated infusion of 40 ng dobutamine/g/min. Heparine was present in the syringes to suppress coagulation. These samples were analyzed twice in a Radiometer ABL805 Flex blood gas analyser (Radiometer, Willich, Germany) at 37°C for pH, pO_2_, pCO_2_, hemoglobin concentration cHb, and oxygen saturation SO_2_, base excess ABE, as well as lactate and glucose concentrations.

### Histology

Left ventricles were cut out from mouse hearts removed after termination of the P-V loop experiments, weighed, then embedded in OCT compound (Tissue-Tek) and frozen at −80°C. Midventricular sections across the entire LV perpendicular to the long axis were stained Masson's trichrome staining and used for determination of cardiac wall thickness by measuring and averaging the thicknesses of the folds of the LV sections.

For fluorescent (immuno)staining, midventricular slices of the left ventricle were embedded in OCT compound and frozen at −80°C. Five micrometer thick cryosections were stained with TRITC-conjugated wheat germ agglutinin (WGA, Sigma-Aldrich) to outline cardiomyocytes. The circumferences of >700 myocytes per LV were traced and digitized to calculate mean cardiomyocyte cross-sectional area. Only cells from fields with circular myocyte shapes (indicative of a transverse section) were analyzed. Fluorescein-labeled GSL I-isolectin B4 (Vector Laboratories, Burlingame, CA) was used to visualize capillaries. Density of cardiomyocyte fibers and of capillaries were counted in about 12 randomly selected areas of 0.03695 *mm*^2^ of each LV. Images were acquired with an Axiovert microscope (Carl Zeiss, Jena, Germany).

### Quantitative real-time PCR (qPCR)

Total RNA was extracted from LV myocardium by TRIzol (Invitrogen) and purified with RNeasy kits (Qiagen, Hilden, Germany). After reverse Transcription (Superscript II, Invitrogen), qPCR of α-MHC (myosin heavy chain), and β-MHC mRNA and SERCA (sarco-endoplasmic reticulum Ca^++^-ATPase) mRNA were performed using the brilliant SYBR Green Mastermix-Kit and the MX4000 multiplex QPCR System from Stratagene. Details as published previously (Korf-Klingebiel et al., 2011).

GAPDH- and PDK1-transcript levels were analyzed on a Rotor-Gene 2000 real-time PCR thermocycler (Qiagen, Hilden, Germany) using Power SYBR Green Supermix (Applied Biosystems, Darmstadt, Germany) according to the manufacturer's instructions. Data were quantified using Rotor-Gene Q Series Software 1.7 (Qiagen). Efficiencies were calculated from the slope of template dilution curves with primers for genes of interest and the reference gene (Rps12), and used for quantification of changes of transcript levels by the ΔΔCt-method. The efficiency of all primer sets was between 95 and 105%. Data were presented as mean ± SE and analyzed by student *t*-test. Primer pairs used were:

Glyceraldehyde-3-phosphate dehydrogenase (Gapdh; GenBank accession no. NM_001289726.1): forward primer: 5′-TGT GTC CGT CGT GGA TCT GA-3′; reverse primer: 5′-CCT GCT TCA CCA CCT TCT TGA-3′.

Pyruvate dehydrogenase kinase 1 (Pdk1; GenBank accession no. NM_172665.5): forward primer: 5′-TGC TAC TCA ACC AGC ACT CC-3′; reverse primer: 5′-TTA ATG ACC TCC ACC ACG TC-3′.

Ribosomal protein S12 (Rps12; GenBank accession no. NM_011295.6): forward primer: 5′-AAG GCA TAG CTG CTG GAG GTG TAA-3′; reverse primer: 5′-AGT TGG ATG CGA GCA CAC ACA GAT-3′.

## Results

### Body and organ weights

**Figure 1:**
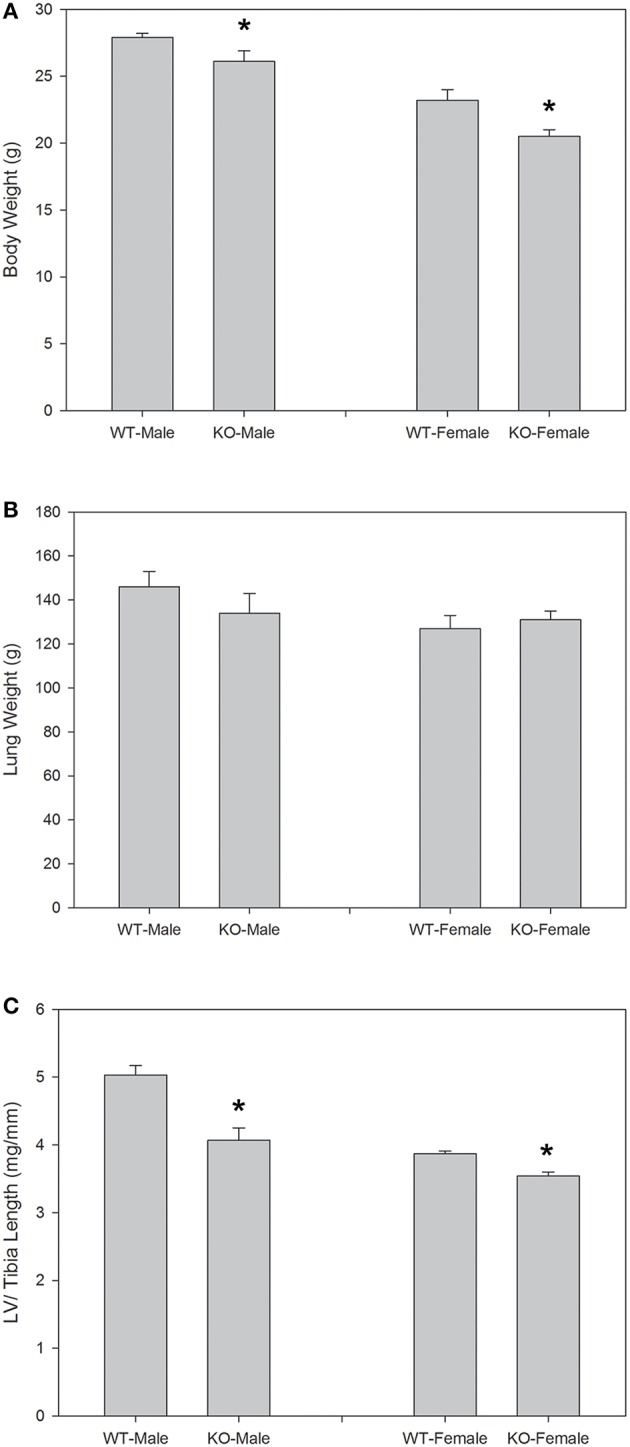
**Body weights (A), lung weights (B), and left ventricular (LV) weights normalized for tibia length (C), for the animals used in the P-V loop measurements**. Sexes are considered separately, and body weights as well as LV weights show significant differences between WT and KO animals for each sex. In the case of LV weights, the significance remains, when sexes are combined. *n* = 7 for both sexes together. ^*^indicates statistically significant difference between KO and WT values.

Figure 1 shows body weights and lung and left ventricular weights from seven wild-type (WT) and seven knockout (KO) mice systemically deficient in AQP1. Each group of mice had three males and four females. It is apparent that body weights are moderately but significantly reduced in the deficient mice, by 6% in males and by 12% in females. Left ventricular weights in Figure 1 were normalized for tibia length; they are reduced in deficient mice by 20% for males, and by 8% for females. Weights of left ventricles (LV) are also reduced in deficient vs. WT mice when not normalized. When normalized for body weight, the differences are present but do not reach statistical significance when the sexes are considered separately. When the sexes are combined, both LV weight per body weight and LV weight per tibia length are significantly lower in deficient vs. WT mice. Thus, there is clear evidence that LV from AQP1-KO mice exhibit a lower weight than those from WT mice. Figure 1 shows in addition, that neither in males nor in females lung weights are affected by the knockout of AQP1. The results of Figure 1 show that the consequences of AQP1-knockout are fundamentally identical in males and females. This holds true also for all other parameters considered in this paper and prompted us in the following to generally consider the combined results from females and males.

### Left ventricular wall thickness of AQP1-deficient mice

**Figure 2:**
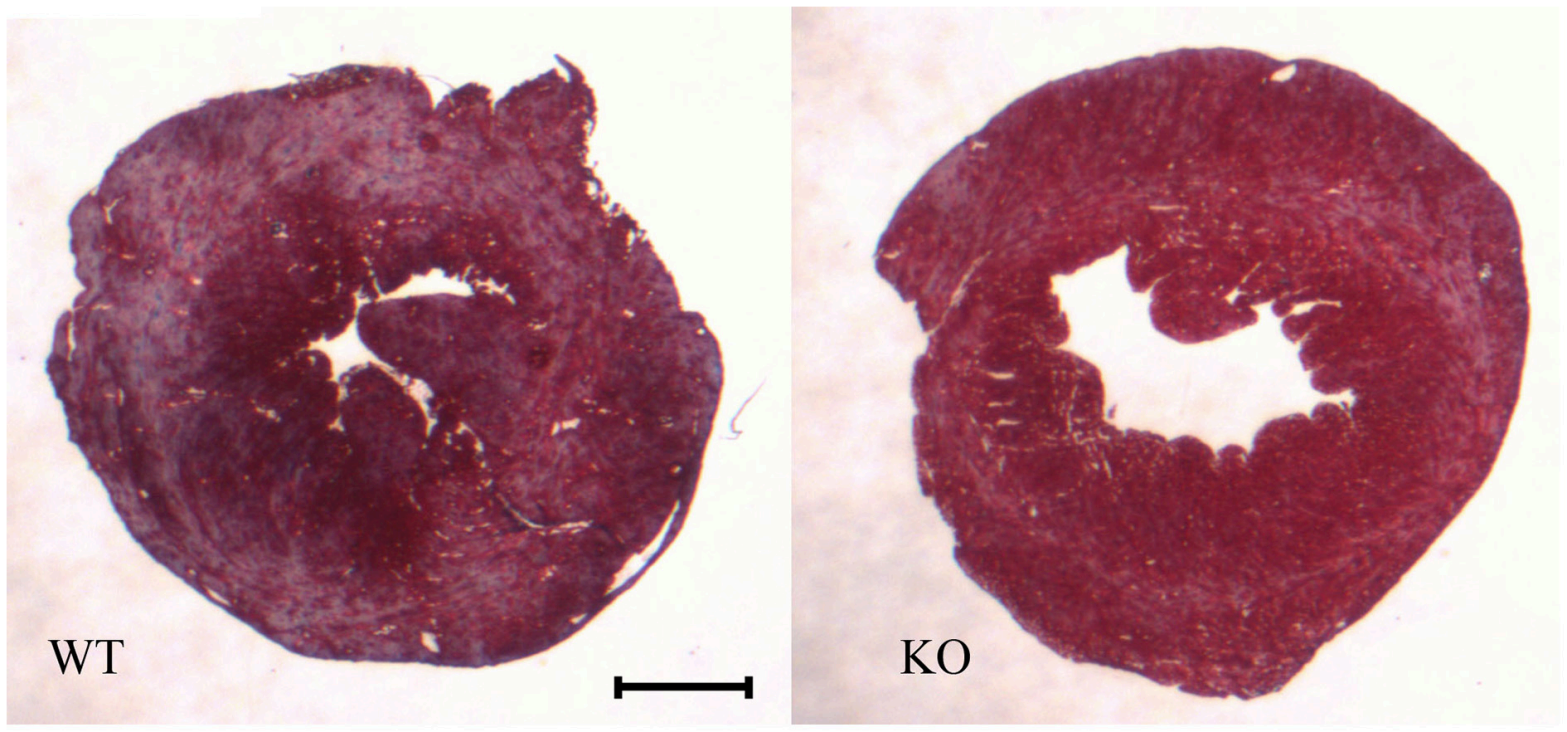
**Heart cross sections, taken perpendicular to the longitudinal heart axis, and stained with Masson's trichrome staining**. Animals are those used in the P-V loop measurements. Bar 1 mm.

Figure 2 shows examples of cross sections of left ventricles of WT and AQP1-KO, which seem to suggest a somewhat thinner wall in the knockout heart. This has been confirmed by an analysis of heart wall thickness in 6 WT and 6 KO hearts (Table 1). Altogether 54 and 50 wall thickness measurements were performed in WT and KO hearts, respectively. We obtain a wall thickness of 1.37 mm (*SE* ± 0.03 mm) in WT hearts, and of 1.22 mm (*SE* ± 0.02 mm) in AQP1-KO hearts. The difference between these values is highly significant (*P* < 0.0001). We conclude that knockout left ventricles exhibit a ~12% thinner wall than WT left ventricles. It appears then that the reduced LV weight of KO hearts is partly or entirely due to a reduced thickness of LV walls.

**Table 1:** **Morphological parameters of left ventricular cardiac muscle of hearts of WT and AQP1-KO mice**.

	**WT (mean ± *SE*)**	**KO (mean ± *SE*)**	***n***	***P***
LV Wall Thickness (mm)	1.37 ± 0.03	1.22 ± 0.02	54/50^a^	< 0.0001
Muscle Fiber Cross Sectional Area (μm^2^)	325 ± 2	277 ± 2	4392/4270^b^	< 0.0001
Muscle Fiber Density (mm^−2^)	3088 ± 58	3327 ± 81	72/70^c^	0.017
Capillary Density (mm^−2^)	3097 ± 46	2827 ± 49	72/70^c^	0.0001
Ratio of Capillary to Muscle Fiber Density	1.01 ± 0.03	0.86 ± 0.04	6^d^	0.02

a *Total number of wall thickness measurements from each group of six hearts, respectively*.

b *Total number of fibers measured in the six hearts of each group*.

c *Number of 0.03695 mm^2^ areas counted in cardiac tissue in each of the two groups of six hearts*.

d *Ratio calculated from the mean values for each of six hearts per group*.

### Cardiac muscle fibers and capillaries in AQP1-deficient mice

**Figure 3:**
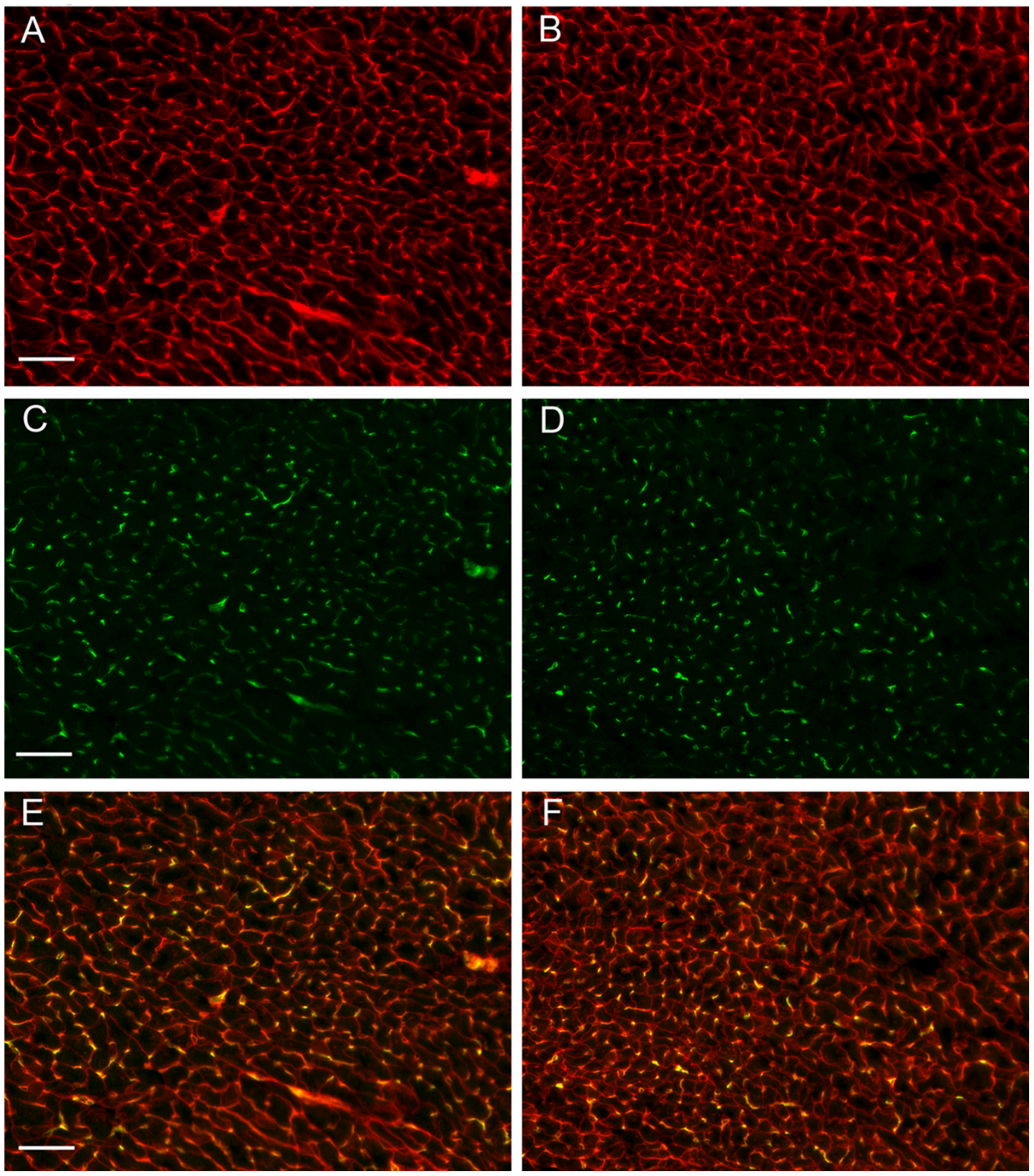
**Sections prepared from cardiac tissue taken from the animals used for PV-Loop measurements. (A,B)** myofiber staining, **(C,D)** capillary staining, **(E,F)** overlay of **A,C, and B,D**, respectively. **A,C,E** are from WT hearts, **B,D,F** are from AQP1- KO hearts. Bar length 50 μm.

Figures 3A,B show examples of muscle fiber staining, Figures 3C,D of capillary staining, and Figures 3E,F show overlays of fiber and capillary staining of WT and KO LV muscle tissue, respectively. Although differences between WT and KO staining patterns are not readily recognizable from these figures, a detailed analysis yields marked differences in several parameters related to muscle fibers and capillaries, as seen in Table 1. The average cross sectional area of WT cardiomyocyte fibers from the left ventricle is 325 μm^2^ (*SE* ± 2 μm^2^), while this value is reduced by 15% to 277 μm^2^ (*SE* ± 2 μm^2^) in AQP1-KO hearts (*P* < 0.0001). In correspondence with this, the density of the muscle fibers is increased from 3088 mm^−2^ (*SE* ± 58 mm^−2^) in WT to 3327 mm^−2^ (*SE* ± 81 mm^−2^) in AQP1-KO mice (*P* = 0.017). Although the number of fibers per area is thus increased by 8% in KO, the number of capillaries per tissue area is decreased by 9%: 3097 capillaries per mm^2^ (*SE* ± 46 mm^−2^) in WT fall to 2827 mm^−2^ (*SE* ± 49 mm^−2^) in KO hearts (*P* = 0.0001). As a consequence of this, the capillary to fiber ratio is 1.01 (*SE* ± 0.03) in WT and is reduced by 15% to 0.86 (*SE* ± 0.04) in AQP1-KO hearts (*P* = 0.02). In summary, the AQP1-KO left ventricles exhibit a reduced muscle fiber thickness in conjunction with an increased fiber density. The latter is associated with a decreased capillary density.

### Cardiac function in AQP1-deficient hearts

**Figure 4:**
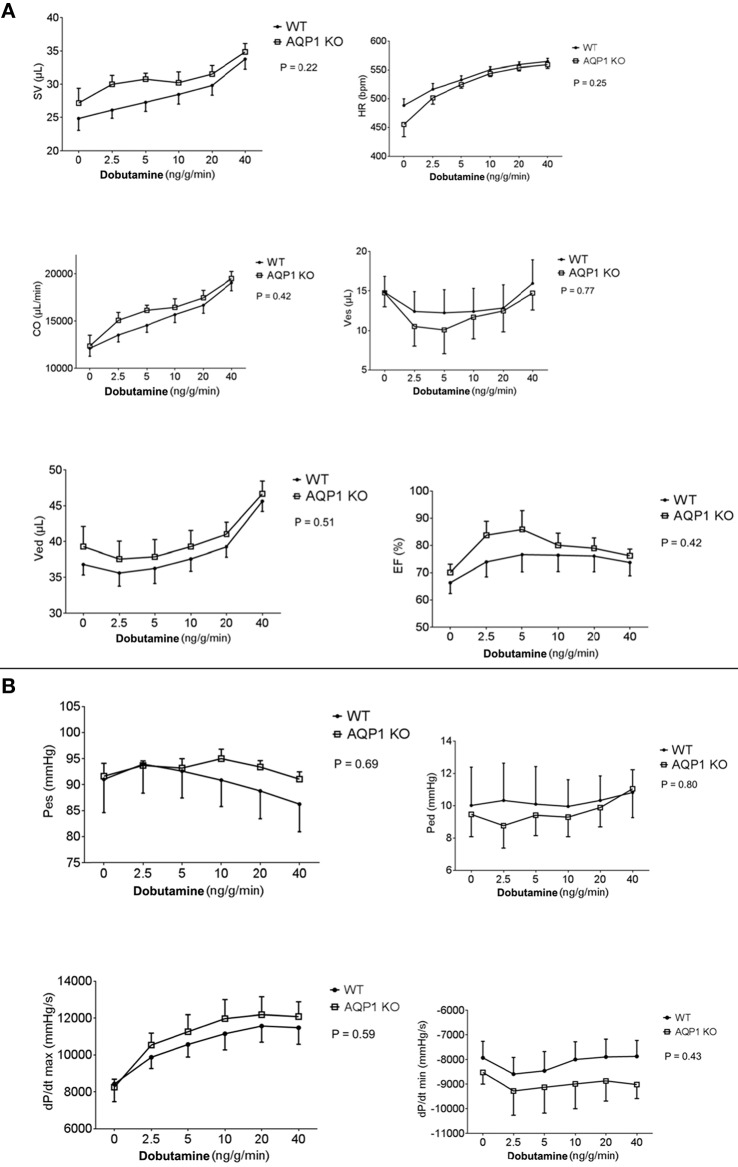
**(A)** Results from PV loop measurements, averaged from seven hearts from both female and male sexes. SV, stroke volume; HR, heart rate; CO, cardia output; Ves, endsystolic volume; Ved, enddiastolic volume; EF, ejection fraction. Infusion of dobutamine was increased stepwise form 0 to 40 ng/g/min. Two-way repeated measures ANOVA indicates no significant difference between curves, indicating that all parameters of WT and KO behave identically. **(B)** Results from PV loop measurements, averaged from seven hearts from both female and male sexes. Pes, end-systolic pressure; Ped, end-diastolic pressure; dP/dt, maximal rate of increase of LV pressure; dP/dt, maximal rate of relaxation of LV pressure. All pairs of curves are not significantly different when tested as described in panel **(A)**.

These studies were essentially obtained by the steady-state LV pressure-volume loop technique. Parameters shown in Figures 4A,B were determined at various infusion rates of dobutamine between 0 and 40 ng/g/min. All parameters given in Figure 4 exhibit no significant difference between WT and AQP1-KO, when subjected to two-way repeated measures ANOVA (Prism 6, GraphPad Software, La Jolla, USA; 2015). This holds, firstly, for the basic functional parameters stroke volume, heart frequency and, consequently, cardiac output. It may be noted that heart rate, even under near-maximal dobutamine stimulation at 40 ng/g/min, does not exceed ~550–600/min (Figure 4A, 1st row, right panel). Under anesthesia, and without cardiac stimulation, heart rates are ~450–500/min, which agrees roughly with the heart rates of ~430/min reported for anesthetized mice without dobutamine stimulation (Hoit et al., 2002). It is noteworthy that, similarly, end-systolic as well as end-diastolic LV volumes and pressures are not different between WT and AQP1-KO hearts, and are influenced by dobutamine in comparable ways (Figures 4A,B). It follows that the ejection fraction of the left ventricle is also identical between WT and AQP1-deficient hearts. Likewise, Figure 4B shows that dP/dtmax and dP/dtmin are not significantly different in WT and AQP1-deficient hearts.

In conclusion, under conditions of anesthesia without as well as with moderate stimulation of the hearts by dobutamine, all functional parameters of AQP1-deficient hearts are identical to WT. The differences in muscle mass and wall thickness between the two mouse types apparently do not affect the function of moderately activated hearts.

### Blood gases and oxygen consumption of anesthetized AQP1-deficient mice

Table 2 shows that in arterial and venous pO_2_, SO_2_, pCO_2_, pH and in base excess, there are no significant differences between WT and AQP1-KO animals. All blood gas values are in the normal range. Hemoglobin concentration of KO mice is slightly but significantly lower than that of WT mice (Table 3), which appears to be at variance with the observation of Montiel et al. (2014), who report identical hematocrits in both strains. Mean corpuscular volume, MCV, is found to be identical in WT and KO mice. Using the arterio-venous SO_2_ differences from Table 2 together with the respective blood hemoglobin concentrations, the cardiac outputs from Figure 4A and the animals' body weights, Table 3 presents the calculated specific oxygen consumptions of WT and AQP1-KO mice under maximal dobutamine stimulation. The values of 0.054 and 0.064 ml O_2_/min/g body weight for WT and KO animals are not significantly different, but are both a factor of 2 or a little less higher than the basal O_2_ consumption of mice of ~0.03 ml/min/g (Rosenmann and Morrison, 1974). This difference is likely due to the stimulatory effect of dobutamine on the animals in the present experiment.

**Table 2:** **Blood gases of AQP1-deficient mice, under 40 ng/g/min dobutamine**.

		**KO ± *SE***	**WT ± *SE***	**n_KO_, n_WT_; P**
1	pO_2_,a (mmHg)	378 ± 16	404 ± 4	6, 8; 0.16
2	pO_2_,v (mmHg)	42.5 ± 2.7	48.0 ± 1.4	6, 10; 0.193
3	ΔpO_2_, a-v (mmHg)	335 ± 13	356 ± 5	6, 8; 0.20
4	SO_2_,a%	100	100	9, 11;
5	SO_2_,v%	53.0 ± 5.6	63.6 ± 3.3	9, 11; 0.13
6	ΔSO_2_, a-v%	47.0 ± 5.6	36.4 ± 3.3	9, 11; 0.13
7	pCO_2_,a (mmHg)	20.1 ± 0.9	20.7 ± 1.1	7, 9; 0.66
8	pCO_2_,v (mmHg)	30.6 ± 1.5	29.7 ± 1.1	9, 10; 0.65
9	ΔpCO_2_, a-v (mmHg)	−10.5 ± 1.9	−8.9 ± 1.3	7, 9; 0.25
10	pHa	7.34 ± 0.017	7.35 ± 0.021	7, 9; 0.80
11	pHv	7.26 ± 0.031	7.29 ± 0.022	9, 11; 0.39
12	ΔpH, a-v	0.08 ± 0.03	0.06 ± 0.02	9, 11; 0.18
13	ABE,a (mmol/l)	−13.2 ± 0.7	−12.8 ± 1.1	8, 9; 0.79
14	ABE,v	−12.3 ± 1.0	−11.7 ± 1.1	9, 11; 0.67
15	Δ ABE, a-v	−0.9 ± 0.7	−1.1 ± 1.2	9, 11; 0.68

*pO_2_, pCO_2_ partial pressures of O_2_ and CO_2_ in blood; SO_2_ blood oxygen saturation; ABE, actual base excess of blood. a, arterial; v, venous; Δ, arterio-venous differences; KO, knockout mice; WT, wildtype mice; SE, standard error of the mean; n, number of determinations. Blood samples were taken from the left and right ventricle, respectively, under dobutamine stimulation at the end of the P-V loop measurements*.

**Table 3:** **Specific oxygen consumption of AQP1-KO and WT mice under 40 ng/g/min dobutamine**.

	**Mean KO ± *SE***	**Mean WT ± *SE***	**n_KO_, n_WT_, P**
CO (μl/min)	19.507 ± 745	19.083 ± 858	6, 7; 0.72
Δ SO_2, a−v_ (%)	47.0 ± 5.6	36.4 ± 3.3	9, 11; 0.13
cHb (g/100 ml)	11.9 ± 0.2	12.9 ± 0.2	7, 6; 0.006
Body wt. (g)	22.9 ± 1.2	25.2 ± 1.1	7, 7; 0.17
VO_2, dob_ (ml/min/g)	0.064 ± 0.012	0.054 ± 0.008	6, 6; 0.49

*CO, cardiac output from P-V loop measurements; Δ SO_2, a−v_, arterio-venous O_2_ saturation difference; cHb, blood hemoglobin concentration; VO_2, dob_, specific oxygen consumption of whole animals under 40 ng/g/min dobutamine infusion, calculated from the other parameters given in the table. KO and WT groups comprise both sexes. The differences in body weights that are statistically significant when sexes are separated (Figure 1), loose significance upon combining sexes, although still apparent. The VO_2, dob_ values shown are nearly a factor of two greater than the basal oxygen consumption of mice of ca. 0.03–0.04 ml/min/g*.

### Expression of some functionally relevant proteins in AQP1-deficient hearts

**Figure 5:**
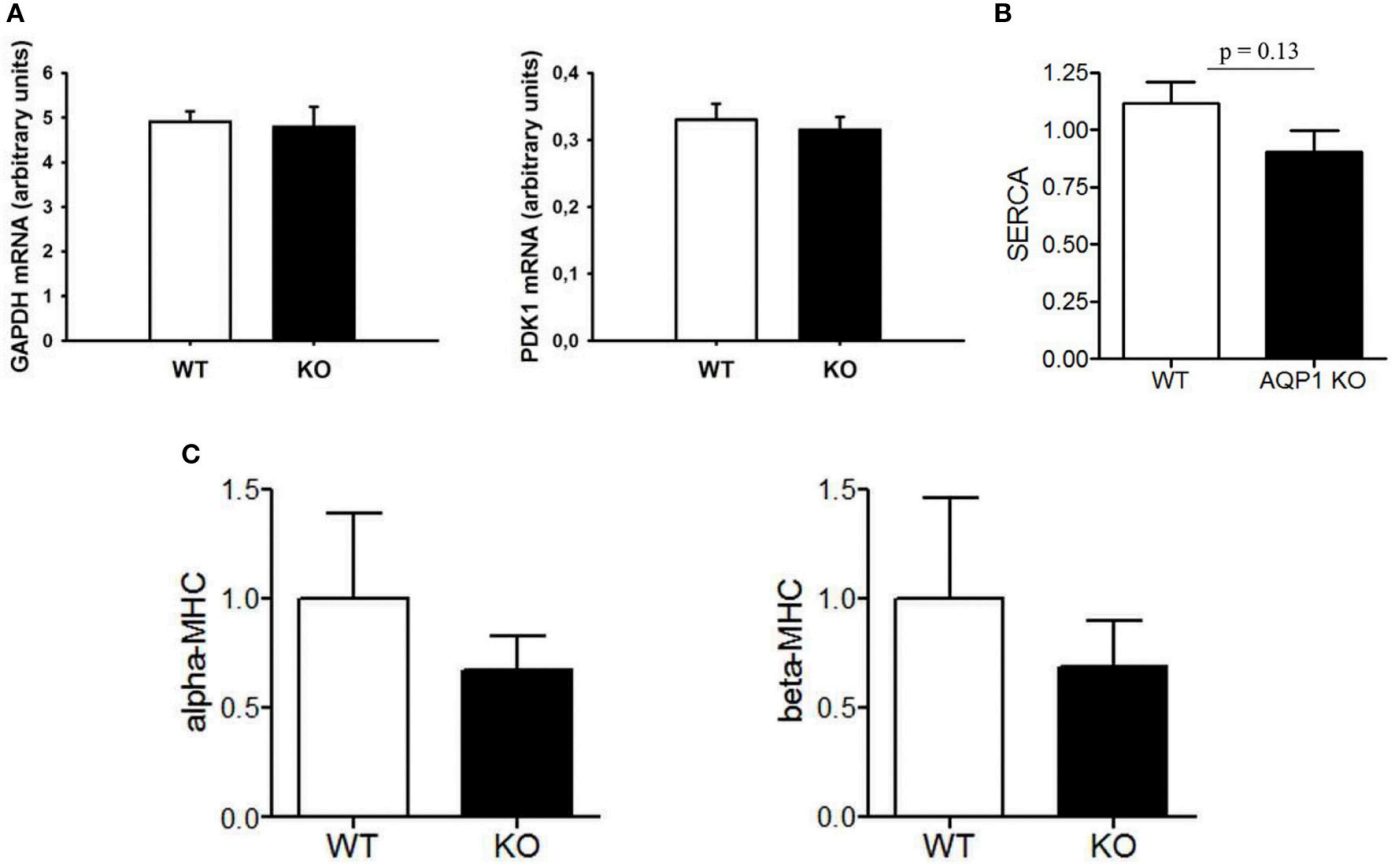
**Analyses of transcripts from LV tissue of the WT and KO mice used in PV loop measurements. (A)**, mRNAs from two HIF-dependent enzymes GAPDH and PDK1; **(B)**, sarco-endoplasmic reticulum Ca^++^-ATPase; **(C)**, α- and β-myosin heavy chain mRNAs.

We have studied two HIF-dependent protein mRNAs to test whether there is evidence for chronic hypoxia in AQP1-deficient hearts. As seen in Figure 5, GAPDH as well as PDK1 mRNA levels are identical in WT and KO hearts, giving no indication of a hypoxic situation in this tissue. Another possible indicator of pathological conditions in mammalian heart is the relation between cardiac expression of α- vs. β-myosin heavy chains, where a decrease in the α form together with an increase in the β form is indicative of a number of pathological situations (Nakao et al., 1997). Figure 5 shows that in the AQP1-deficient hearts there are no significant differences in the expression of the two myosin heavy chain isoforms to that observed in WT hearts. Clearly, there is no increase in β-MHC expression in the KO hearts.

In addition, we asked whether the diminished cardiac muscle mass of KO mice might be functionally compensated by an upregulation of sarcoendoplasmic reticulum ATPase (SERCA) in cardiomyocytes. Figure 5 shows that this is not the case. Thus the normal function of AQP1-deficient hearts is maintained by another mechanism than increased expression of SERCA. This latter finding is in agreement with the unaltered dP/dtmax of AQP1-deficient hearts (Figure 4B).

## Discussion

### Morphological characteristics of the AQP1-deficient mouse heart

Two basic findings for AQP1-KO mice shown in Figure 1 confirm previous observations: body weight of AQP1-KO mice is reduced by 6–12% compared to WT (Figure 1A). Similar body weight reductions have been reported by Schnermann et al. (1998). Also, left ventricular mass (normalized to tibia length) is significantly reduced by 8–20% in males and females (Figure 1C), a finding qualitatively obtained also by Montiel et al. (2014). A third result of the present paper, the reduced cardiac muscle fiber cross sectional area in KO hearts (Table 1) that goes along with a diminished heart muscle mass, has also been reported by Montiel et al. (2014). However, three further findings of this study that are of major functional importance and are shown in Table 1, have not been reported before or are at variance with the observations of Montiel et al. (2014): (1) the reduced LV wall thickness of AQP1-deficient hearts, (2) the reduced capillary density in LV muscle, and (3) the even more pronounced reduction of the ratio capillary to muscle fiber density compared to WT hearts. In contrast to findings (2) and (3), Montiel et al. (2014) have reported an unaltered capillary to myocyte ratio, implying that, like muscle fiber density, capillary density is even increased in AQP1-KO hearts. The reason for the discrepancy is not clear. In view of the present result of a reduced LV wall thickness, we conclude that—at least the present—AQP1-deficient hearts exhibit a reduced muscle mass in combination with thin ventricle walls. In conjunction with the finding of normal end-diastolic and end-systolic LV volumes as shown in Figure 4 and discussed below, the emerging picture of the AQP1-deficient heart is that of a heart of normal volume but reduced muscle mass due to decreased thickness of ventricle walls. The underlying cause of the reduced muscle mass may solely be the reduced muscle fiber size (Table 1), while the total number of muscle fibers per heart, although not known, may be normal. Whereas muscle fiber density is increased due to the fibers' smaller size, the absolute density of capillaries is reduced. This implies that the diffusion distances for O_2_, substrates and metabolites are increased. In summary, the AQP1-deficient heart seems to be at a disadvantage compared to the WT heart due to its thinner ventricle walls with diminished muscle mass and to its greater tissue diffusion resistance toward O_2_.

### Cardiac function and gas exchange in anesthetized WT and KO animals

In the following we will discuss the absolute values of cardiac and respiratory parameters of anesthetized mice parameters and the effects of dobutamine upon them.

#### Cardiac function

As apparent from Figure 4, none of the parameters obtained in the P-V loop measurements was significantly different between WT and KO mice. Some parameters clearly increase with increasing dobutamine concentration, e.g., stroke volume (SV), heart rate (HR), cardiac output (CO), end-diastolic volume (Ved), and dP/dtmax. However these, as all other parameters, depend on dobutamine in a manner indistinguishable between WT and KO mice. We conclude that anesthetized KO mice, even under dobutamine stimulation, exhibit an entirely normal cardiac function. This holds in spite of the reduced cardiac muscle mass and capillarisation as discussed above.

How do the numbers of Figure 4 compare to those of conscious resting mice? With microsphere and dilution techniques, Barbee et al. (1992) have obtained a **heart rate** of 652 min^−1^ for conscious mice in a state after recovery from Avertin anesthesia and in conditions restraining their mobility. Janssen et al. (2002) have reported a heart rate of 600 min^−1^ in conscious resting mice. Both figures are clearly higher than our values obtained under anesthesia without as well as with dobutamine stimulation (Figure 4A, 1st row, 2nd panel). It may be noted that other authors report a resting heart rate of only 450–550 min^−1^ in mice in a state after waking up from anesthesia (Hoit and Walsh, 2002, p. 283; Kramer et al., 1993). No matter whether we consider resting heart rate in conscious mice to be 652, 600, or 450–550 min^−1^, we can conclude that all heart rates of anesthetized mice seen in Figure 4A, even in the presence of the highest concentration of dobutamine, do not exceed the range of values observed in conscious resting animals. Mice exercising on a treadmill, on the other hand, reach heart rates up to about 800 min^−1^ (Hoit and Walsh, 2002, p. 284; Segrem and Hart, 1967; Kramer et al., 1993; Desai et al., 1997; Schuler et al., 2010, and personal communication by Beat Schuler). So, dobutamine in anesthetized mice does stimulate heart rate, but not to levels outside the range of resting levels of conscious animals, and its effect remains far below of what is seen in exercising mice. The reason for this is the drastic depressive effect of anesthesia on heart rate as demonstrated for example by Barbee et al. (1992).

Likewise, the **stroke volumes** seen in Figure 4A are similar to the value of ~30 μl reported for the resting mouse by Janssen et al. (2002). Also the **cardiac outputs** seen in Figure 4A are all in the range of values reported for the resting mouse, 16 ml/min by Barbee et al. (1992) and 20 ml/min by Janssen et al. (2002). Again, dobutamine does not raise these parameters to levels above the range of resting values in unanesthetized mice. In conclusion, all three cardiac parameters in this study remain under anesthesia in the range of resting values of conscious resting animals even when dobutamine stimulation is applied.

#### Blood gases

All values of Table 2 are similar to what is expected for humans and other mammals respiring 95% O_2_/5% CO_2_. As in the case of the parameters of cardiac function, there are no significant differences in blood gas values of anesthetized dobutamine-treated WT vs. KO animals (Table 2). Specifically, arterial pO_2_ and SO_2_ are identical in both types of animals, suggesting that pulmonary O_2_ exchange is not affected by the lack of AQP1. It should be noted that the high inspiratory pO_2_ (corresponding to 95% O_2_) can render a moderate pulmonary diffusion problem or an unequal distribution of ventilation/perfusion invisible in terms of their effect on SO_2_, but their effect on pO_2_,a might remain visible. In addition, mixed venous pO_2_ and SO_2_ are not significantly different, suggesting that peripheral O_2_ exchange is also unaffected. However, in both independent measurements of pO_2_,v and SO_2_,v there is a tendency toward lower values in KO compared to WT animals. It may be speculated that this points to a need for enhanced O_2_ extraction in the periphery of KO animals, which would be necessary when either VO_2_ is higher and/or CO is lower in KO than in WT animals, two possibilities we can both exclude under resting conditions on the basis of the present data (Table 3, Figure 4). Clearly, this finding excludes a markedly increased resistance for transfer of O_2_ from blood to tissues in animals lacking AQP1, because this would be expected to lead to rather enhanced values of pO_2_,v and SO_2_,v. An identical conclusion is arrived at when considering the exchange of CO_2_. Arterial pCO_2_ is identical between KO and WT animals, and likewise mixed venous pCO_2_ values are identical. This suggests that pulmonary as well as peripheral CO_2_ exchange is unaffected by the absence of AQP1 in animals under “resting conditions”. It may be noted that Yang et al. (2000) and Swenson et al. (2002) in studies of pulmonary CO_2_ exchange under “resting conditions” also found no evidence for a role of aquaporin.

#### Oxygen consumption

Table 3 shows that, firstly, oxygen consumption of anesthetized mice at maximal dobutamine stimulation is around 0.06 ml/min/g. This is twice or a little less the basal oxygen consumption of mice of 0.03–0.04 ml/min/g (Hoit and Walsh, 2002, p. 285 Segrem and Hart, 1967; Rosenmann and Morrison, 1974; Heldmaier and Neuweiler, 2004). This slightly elevated level is likely due to the maximal dobutamine stimulation prevailing when the parameters cardiac output and blood oxygen saturation, which are used to calculate VO_2_, are measured here. This is in agreement with the observation of Rosenmann and Morrison (1974) on the effect of adrenergic stimulation on VO_2_ of mice. Secondly, Table 3 shows that, at this moderately elevated level of VO_2_, aquaporin-1 deficiency has no significant effect on VO_2_. Since conscious mice due to their normal level of physical activity will exhibit a similar VO_2_ (= “resting VO_2_”; Segrem and Hart, 1967; and own unpublished observations), we can expect that AQP1-deficient laboratory mice moving normally in their cages will have no limitation of oxygen supply.

#### Summary of cardiac and respiratory functions in anesthetized AQP1-KO mice

Although AQP1 is present in capillaries of most tissues such as skeletal and cardiac muscle and the lung (Nielsen et al., 1993; Verkman, 2007; Rutkovskiy et al., 2013), the present data indicate that it does not affect O_2_ and CO_2_ exchange in these organs under conditions of moderate VO_2_. It should be noted that in skeletal and heart muscle and in the lung other AQP isoforms are strongly expressed, AQP4 in the plasma membranes of skeletal and cardiac myocytes and AQP5 in the plasma membrane of alveolar epithelia (Verkman, 2007; Rutkovskiy et al., 2013). Both AQP5 and AQP4 are similarly good conductors of CO_2_ as AQP1 (Musa-Aziz et al., 2009) and might substitute for AQP1 in its possible role in gas transfer. Additionally we have shown previously that a role of gas channels in gas transfer would become apparent only under maximal workload and metabolism but not under resting conditions (Endeward et al., 2014). In the lung, for example, this implies that at rest and moderate workload CO_2_ exchange remains perfusion-limited, and becomes diffusion-limited only under conditions of maximal exercise (Endeward et al., 2008). Thus, it is in line with these previous studies that in this paper we find no indication of an altered gas exchange in anesthetized AQP1-deficient animals.

Similarly, we find here that cardiac function under the present conditions of moderately elevated VO_2_ is normal. This may seem surprising in view of the thinner LV walls and the reduced cardiac muscle mass in AQP1-KO animals. We propose that this may result from a compensation mediated by the Frank-Starling mechanism. Thinner walls will be associated with an increased compliance ΔV/ΔP of the ventricle. Indeed, end-diastolic volume/end-diastolic pressure is on average 4.2 μl/mmHg for KO hearts and 3.7 μl/mmHg for WT hearts in the absence of dobutamine, indicating a greater ventricular compliance in KO hearts, although the above difference does not reach statistical significance. When compliances are different and intraventricular pressures are identical between WT and KO mice, as apparent from Figure 4, the tension and length of the individual muscle fibers are expected to be greater in KO than in WT hearts. Thus, although KO hearts possess less muscle mass, their force production may be equal to WT hearts because of the increased strain of their fibers. This would explain the normal stroke volume generated by KO hearts under resting conditions. An analogous conclusion is arrived at by considering Laplace's law. While this mechanism obviously is able to maintain a normal heart function at moderate workloads, it must be expected that the KO heart will exhibit a diminished maximal performance at very high workloads. In this context, it is noteworthy that Boron (2010) has observed that AQP1-KO mice exhibit a 40% reduced spontaneous physical activity on a running wheel compared to WT mice.

Figure 5 strongly supports this view of an entirely normal heart function in AQP1-KO mice under the conditions they are kept at in the laboratory. The unaltered expression of the mRNA of HIF-dependent enzymes GAPDH and PDK1 suggests that there is no tissue hypoxia in the heart under the conditions of the minor physical activity mice exhibit in their cages. Similarly, the ratio of α- to β-MHC is unaltered, so this again does not suggest a pathological condition of the heart. Lastly, there is clearly no up-regulation of cardiac SERCA, which could occur in an attempt to increase the contractility of the reduced total muscle cross-sectional area of KO hearts. All three parameters give no indication of pathological conditions or adaptations to pathological conditions that could be envisaged in these hearts. We conclude that AQP1-KO mouse hearts under “resting conditions” seem to be entirely up to their tasks. Limitations are only expected to become apparent under conditions of maximal workloads that do not occur under normal conditions in laboratory mice.

It may be noted that in the case of AQP1-deficient humans neither size and wall thickness of the hearts have been described, nor have results of exercise testing of these patients been reported (King et al., 2001; Verkman, 2008).

### A possible cause of the diminished muscle mass of AQP1-deficient hearts

The major morphological alterations of the AQP1-deficient heart that have been described in this study are: reduced cardiac muscle mass, reduced LV wall thickness, reduced cross-sectional area of ventricular myofibers, reduced ratio of capillaries to myofibers, and reduced absolute density of capillaries in LV tissue. Is there one underlying primary mechanism that leads to these alterations? We have no experimental evidence on such a mechanism, but we can speculate on the basis of two established properties of AQP1 as mentioned above, that of a gas channel and that of an essential element in angiogenesis.

If cardiac tissue is deprived of a channel for CO_2_, possibly also for O_2_, could some of the morphological alterations just listed be envisaged as direct consequences? If the diffusion resistance of the capillary wall (and the sarcolemma) for O_2_/CO_2_ is increased due to the lack of AQP1, the only possible adaptation during heart development would seem to be an increase in capillary density, which would reduce the diffusion distances and thus compensate for the increase of membrane resistances toward CO_2_/O_2_. Table 1 shows that these hearts instead show a decrease in capillary density, which will even impair gas diffusion between capillaries and muscle mitochondria. Thus, it appears unlikely that the observation of Table 1 represents a direct consequence of the lack of AQP1 in its function as a CO_2_/O_2_ channel.

A distinct deviation of the present results from those of Montiel et al. (2014) is our observation of a reduced capillary density in heart muscle. This opens up the possibility that the reduced size of left ventricular cardiomyocytes and wall thickness is the direct consequence of an impaired capillarisation due to the absence of AQP1. We will discuss two possible mechanisms that could link capillarisation to the expression of AQP1.

Firstly, as stated above, another gas proposed to be conducted by AQP1 is NO (Herrera et al., 2006; Herrera and Garvin, 2007). These authors have shown NO flux out of endothelial cells and into smooth muscle cells to occur via AQP1. Furthermore, they have reported that NO-dependent relaxation in thoracic aortas is impaired in AQP1-KO mice, although this latter finding has not been confirmed by Montiel et al. (2014). Since NO has been shown to play a central role in vascular development (Yuan and Kevil, 2016), it would be conceivable that these effects of NO are also mediated by AQP1. Thus one might speculate that AQP1 deficiency inhibits the stimulating effect of NO on vascular growth. There is no experimental evidence for such a pathway at present but it could, in addition to the mechanism proposed below, provide an explanation of the reduced capillary density we find here in AQP1-deficient cardiac muscle.

Secondly, aside from the conduction of NO by AQP1, aquaporin's function as a water channel may provide another explanation for an impaired angiogenesis. AQP1 is widely accepted to play a central role in angiogenesis and, generally, in cell migration (Papadopoulos et al., 2008). The molecular mechanism of this role is not fully understood, but has been speculated to involve AQP1-mediated water fluxes across the membranes of the migrating cells (Papadopoulos et al., 2008). Cell migration is essential for angiogenesis, as well as for several other biological processes like wound healing, tumor spread, and organ regeneration. As mentioned above, AQP1 is expressed in most blood capillaries, and it has been shown during the last few years that endothelial AQP1 is involved in angiogenesis in a multitude of tumors (Saadoun et al., 2005; Yang et al., 2006; Otterbach et al., 2010; López-Campos et al., 2011; Nicchia et al., 2013; Zou et al., 2013; Esteva-Font et al., 2014). AQP1 is also involved in angiogenesis in other tissues such as in heart tissue after myocardial infarction (Ran et al., 2010), in neovascularization of the cirrhotic liver (Huebert et al., 2010; Yokomori et al., 2011), and in tube formation of human retinal vascular endothelial cells (Kaneko et al., 2008). Thus, it seems well-established that AQP1 is essential in capillary growth. If this occurs in the same way during development of the heart, we can predict that lack of AQP1 will impair capillarisation of heart tissue, which would also explain the diminished capillarisation seen for the adult KO heart in Table 1. Reduced capillarisation of cardiac muscle could be the primary event that leads to all other abnormalities of AQP1-KO hearts compiled in Table 1: It has been shown (Tirziu et al., 2007) that growth of cardiomyocytes as well as of total heart weight is regulated by capillary density. Stimulating capillary growth caused cardiac hypertrophy. Vice versa, in the present case inhibition of capillary growth by lack of AQP1 can be expected to impair growth of the individual cardiomyocytes as reported in Table 1. If the total number of fibers per heart remains unaltered, this will lead to reduced wall thickness and cardiac muscle mass. It is thus conceivable that by this chain of events, lack of AQP1 during heart development causes the reduced wall thickness and muscle mass we observe here. This should lead to a reduced maximal stroke volume and maximal cardiac output of the AQP1-deficient heart, which would limit maximal physical performance of these mice. This cannot be demonstrated under the conditions of the anesthetized mouse, and other approaches than those used here will be necessary to achieve this.

## Author contributions

SA has performed histological evaluations and together with GG the blood gas measurements, YW has performed PV loop measurements and qPCR of MHCs and SERCA, JM has performed PCR of HIF-dependent proteins, GG and VE have contributed the concept of the study and have written the first draft of the paper.

### Conflict of interest statement

The authors declare that the research was conducted in the absence of any commercial or financial relationships that could be construed as a potential conflict of interest.
